# Serum uric acid and risk of cardiovascular mortality: a systematic review and dose-response meta-analysis of cohort studies of over a million participants

**DOI:** 10.1186/s12872-019-1215-z

**Published:** 2019-10-15

**Authors:** Fatemeh Rahimi-Sakak, Mahsa Maroofi, Jamal Rahmani, Nick Bellissimo, Azita Hekmatdoost

**Affiliations:** 1 Student Research Committee, Faculty of Nutrition and Food Technology, Shahid Beheshti University of Medical Sciences, Tehran, Iran; 2 School of Nutrition, Ryerson University, Toronto, Ontario Canada; 3 Department of Clinical Nutrition and Dietetics, Faculty of Nutrition Sciences and Food Technology, National Nutrition and Food Technology Research Institute, ShahidBeheshti University of Medical Sciences, Tehran, Iran

**Keywords:** Uric acid, Serum uric acid, Cardiovascular diseases mortality, CVD mortality, Longitudinal, Cohort

## Abstract

**Background:**

Cardiovascular disease (CVD) is the leading cause of death worldwide. Some studies have suggested anassociation between serum uric acid levels and cardiovascular mortality; however, the results have not been summarized in a meta-analysis.

**Methods:**

A comprehensive search of all related studies until April 2018was performed in MEDLINE/PubMed and Scopus databases DerSimonianand Laird random-effects models were used to combine hazard ratios (HRs) with 95% confidence intervals (CIs). Dose-response analysis was also carried out.

**Results:**

Thirty-two studies containing forty-four arms with 1,134,073 participants reported association between uric acid and risk of CVD mortality were included in our analysis. Pooled results showed a significant positive association between uric acid levels and risk of CVD mortality (HR 1.45, 95% CI 1.33–1.58, I^2^ = 79%). Sub-group analysis showed this relationshipwasstronger in women compared to men. Moreover, there was a significant non-linear association between uric acid levels and the risk of CVD mortality (r = 0.0709, *p* = 0.001).

**Conclusion:**

Our analysis indicates a positive dose-response association between SUA and CVD mortality risk.

## Background

Cardiovascular diseases (CVD) are the first leading cause of death worldwide [1]. This might be due to an increased incidence of chronic diseases such as obesity, diabetes, hypertension, dyslipidemia, and hyperuricemia [2]. Uric acid (UA) is considered the ultimate product of purine metabolism in humans. Evidence suggests that increased levels of serum uric acid (SUA) are associated with the development of hypertension, coronary heart disease (CHD), cardiovascular stroke, cerebrovascular accidents (CVA), and cardiovascular disease [3].SUA concentrations can reflect the amount of purine intake from the diet, inborn purine metabolism, changes in UA secretion (reduced glomerular filtration and tubular secretion, or increased tubular reabsorption), and intestinal degeneration [4]. The relationship between SUA and CVD was first reported more than 50 years ago, and several epidemiological studies were conducted to assess the association between hyperuricemia (HU) and CVD [5]. Although many studies have been conducted assessing the relationship between UA and CVD, there is disagreement about this relationship [6]. These controversies are due to the dual effect of UA in the body [7]. The atherogenic effects of UA include induction of oxidative stress in cells, which reduces the bioavailability of nitric oxide - associated with the activity of platelets and endothelial cells and the differentiation of smooth muscle cells in the vascular system. On the other hand, UA can also have antioxidant properties that can prevent atherosclerosis and improve endothelial function [8].

To gain a greater understanding of the prognostic value of SUA for future clinical decision making, we conducted a meta-analysis of prospective cohort studies with dose– response analysis to determine the relationship between SUA and CVD mortality.

## Methods

This meta-analysis conducted by following the Meta-analysis of Observational Studies in Epidemiology study guidelines (MOOSE) [9]. A comprehensive literature search was carried out by two reviewers (MM) and (FRS) independently on PubMed/MEDLINE (https://www.ncbi.nlm.nih.gov/pubmed/) and Scopus (https://www.scopus.com/search/) databases up to April 2019 without any time restriction. Following keyword was followed for systematic search: in PubMed/MEDLINE: (((“Uric Acid”[Mesh] OR uric acid [Title/Abstract]) OR serum uric acid [Title/Abstract]) AND ((((“cardiovascular disease mortality”[Title/Abstract] OR “cardio vascular mortality”[Title/Abstract]) OR “cardiovascular mortality”[Title/Abstract]) OR “CVD mortality”[Title/Abstract]) OR CVD-mortality [Title/Abstract])) AND (((prospective [Title/Abstract] OR longitudinal [Title/Abstract]) OR follow-up [Title/Abstract]) OR cohort [Title/Abstract]), in Scopus: ((TITLE-ABS-KEY (prospective) OR TITLE-ABS-KEY (longitudinal) OR TITLE-ABS-KEY (follow-up) OR TITLE-ABS-KEY (cohort))) AND ((TITLE-ABS-KEY (uric AND acid) OR TITLE-ABS-KEY (serum AND uric AND acid))) AND ((TITLE-ABS-KEY (cardiovascular AND disease AND mortality) OR TITLE-ABS-KEY (cardiovascular AND mortality) OR TITLE-ABS-KEY (cardio AND vascular AND mortality) OR TITLE-ABS-KEY (cvd AND mortality) OR TITLE-ABS-KEY (cvd-mortality))).

### Exclusion and inclusion criteria and data extraction

Non-English articles, reviews papers, editorials, non CVD mortality, non-human studies, in vitro research, case reports, and letters without sufficient data were excluded. Studies that met the following inclusion criteria were included in this meta-analysis:

1) Cohort study design with CVD mortality outcome.

2) Hazard ratio (HR) and the corresponding 95% confidence interval (CI) of CVD mortality were reported based on uric acid levels.

The first author, publication year, country, study design, number of participants, mean age, gender, years of follow-up, hazard ratios and 95% CIs of CVD mortality information extracted from included studies.

### Statistical analysis

The STATA 14.0 statistical software (Stata Corporation, College Station, Texas, USA) was used for statistical analyses. Combined results of hazard ratio of CVD mortality conducted by Random-effects model [10]. *P* < 0.10 and I2 < %50 were considered as heterogeneity detection among included studies. In order to find source of heterogeneity, subgroup analysis was conducted based on gender, whereas meta-regression analysis was conducted based on follow-up years, age of participants and HR of CVD mortality. Non-linear association was examined by modeling concentration level using restricted cubic splines [10]. The publication bias among included studies evaluated by Funnel plot, Begg’s test, and Egger’s regression test.

## Results

### Literature search

**Fig. 1:**
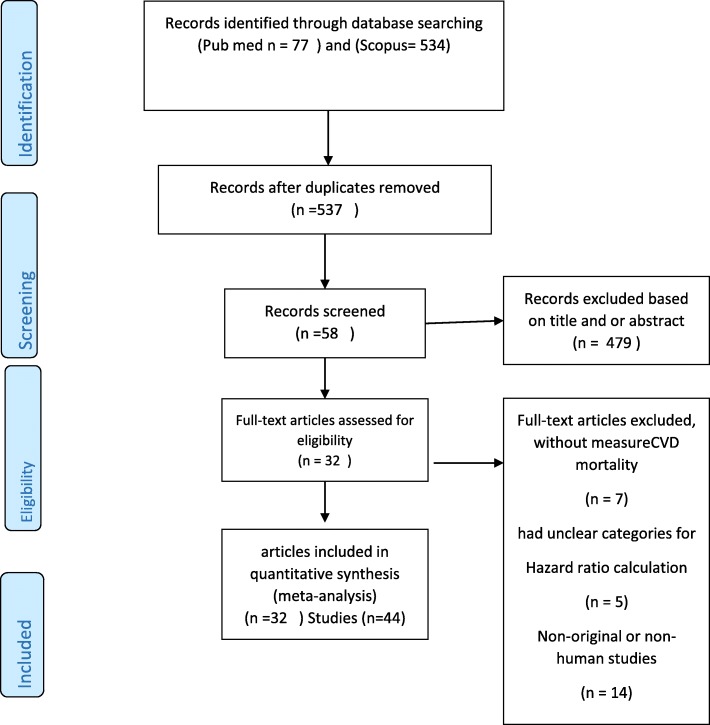
Flow chart of studies reviewed

A flow chart of included studies is shown in Fig. 1. In the primary search 611 records were identified, after excluding 74 duplicates studies and 479 irrelevant studies from title and abstract screening, 58 studies remained for full text screening. After reviewing the full text, 26 studies were excluded because they did not meet the inclusion criteria. Finally, 32 studies, containing 44 arms and 1,134,073 participants, were included in the meta analysis.

**Fig. 2:**
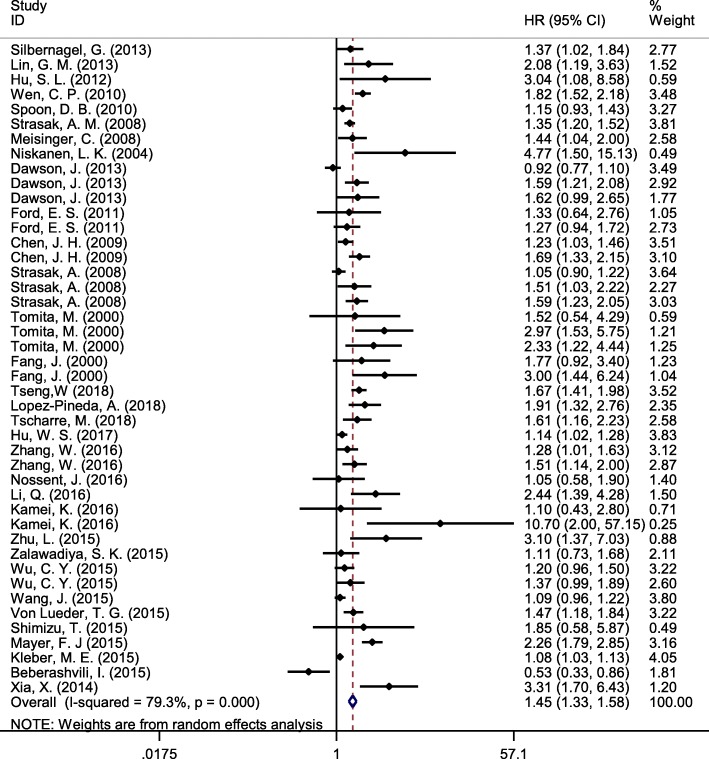
The forest plot between highest versus lowest categories of serum uric acid and cardiovascular disease mortality

### Study characteristics and quality assessment

Table 1 shows characteristics of included studies. Studies were published between 2000 and 2018. The mean age of participants was 55.9 years and the mean duration of follow-up was 9 years from 1 to 18 years. Seventeen studies were performed in Asia and Australia [11–27], and fifteen in Europe and America [28–42]. Twenty-two arms were conducted in both genders, eight in women, and fourteen in men.

**Table 1:** Baseline Characteristics of Included Studies in the Meta-analysis

Studies	Author	Year	Country	Follow up (year)	Sex (1-women, 2-men, 3-both)	Patients, n
1	Silbernagel, G.	2013	Germany	7.3	3	3245
2	Lin, G. M.	2013	Taiwan	3.2	3	1054
3	Hu, S. L.	2012	Taiwan	10	3	1093
4	Wen, C. P.	2010	Taiwan	8.5	3	230,508
5	Spoon, D. B.	2010	USA	2	3	1916
6	Strasak, A. M.	2008	Austria	21	1	28,613
7	Meisinger, C.	2008	Germany	11.7	2	3604
8	Niskanen, L. K.	2004	Finland	11.9	2	1423
9	Dawson, J.	2013	Scotland	10	1 and 2	6984
10	Ford, E. S.	2011	USA	14	3	13,802
11	Chen, J. H.	2009	Taiwan	8.2	1 and 2	90,393
12	Strasak, A.	2008	Austria	13.6	2	83,638
13	Tomita, M.	2000	Japan	5.4	2	49,413
14	Fang, J.	2000	USA	16.4	1 and 2	5926
15	Tseng,W	2018	Taiwan	5.8	3	127,771
16	Lopez-Pineda, A.	2018	Spain	3	3	1119
17	Tscharre, M.	2018	Austria	5.5	3	1215
18	Hu, W. S.	2017	china	8.8	3	381,963
19	Zhang, W.	2016	Japan	23	1 and 2	36,313
20	Nossent, J.	2016	Australia	15	3	3475
21	Li, Q.	2016	china	3.9	3	1799
22	Kamei, K.	2016	Japan	8	1 and 2	3487
23	Zhu, L.	2015	china	12	3	588
24	Zalawadiya, S. K.	2015	USA	14.5	3	11,009
25	Wu, C. Y.	2015	Taiwan	5	1 and 2	77,541
26	Wang, J.	2015	china	6	3	2585
27	Von Lueder, T. G.	2015	Norway	2.7	3	12,677
28	Shimizu, T.	2015	Japan	1.67	3	424
29	Mayer, F. J	2015	Austria	6.3	3	959
30	Kleber, M. E.	2015	Germany	10	3	3315
31	Beberashvili, I.	2015	Israel	2	3	261
32	Xia, X.	2014	China	2.1	3	985

### Main results of the meta-analysis

**Fig. 3:**
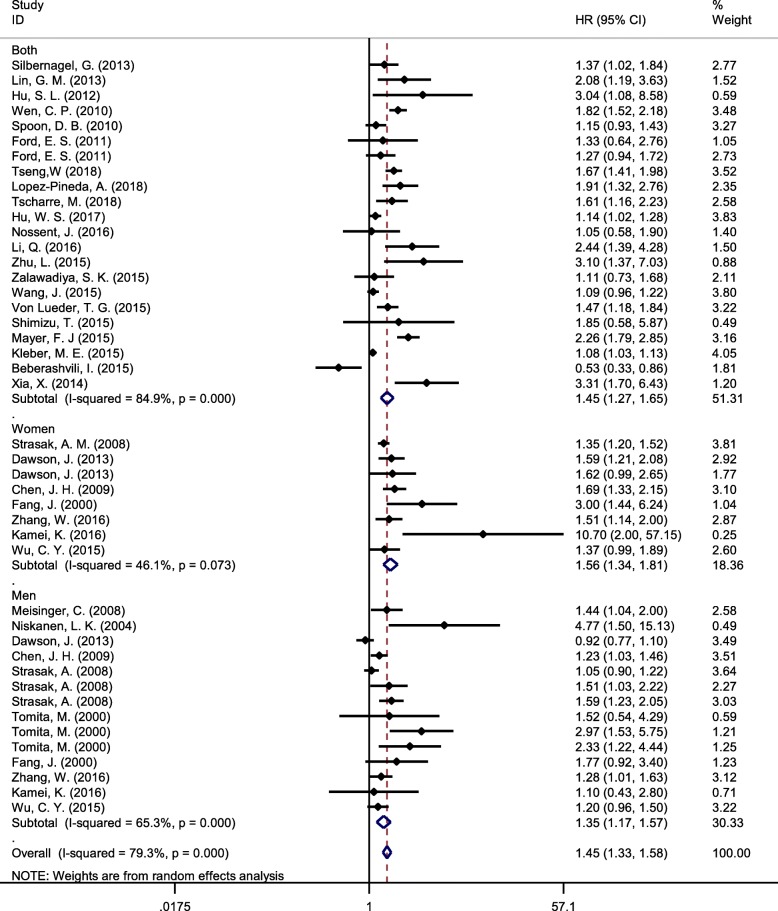
The forest plot between highest versus lowest categories of serum uric acid and cardiovascular disease mortality based on gender

In 44 arms, pooled results from the random effects model showed a positive association between SUA and risk of CVD mortality in highest versus lowest category of SUA (HR 1.45, 95% CI 1.33–1.58, I2 = 79%) (Fig. 2). Subgroup analysis based on a gender showed a stronger relationship in women compared with men (Fig. 3). Meta-regression analysis did not show any significant relationship between SUA and risk of CVD mortality based on participant age (*p* = 0.86) or duration of follow-up(*p* = 0.44).

### Dose-response analysis

**Fig. 4:**
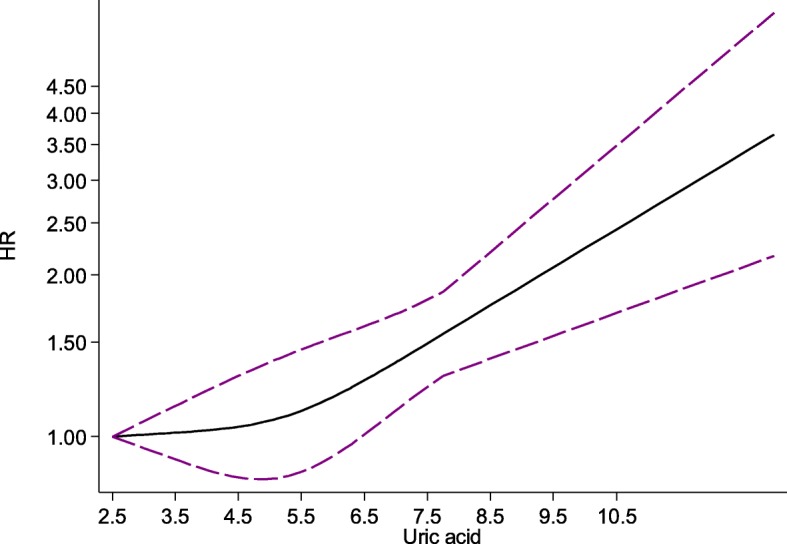
Dose-response association between serum uric acid (mg/dL) and cardiovascular disease mortality (HR)

The pooled HR from the random-effects dose-response model of included studies showed a significant positive association between SUA and CVD mortality (r = 0.0709, *p* = 0.001) (Fig. 4).

### Publication bias

**Fig. 5:**
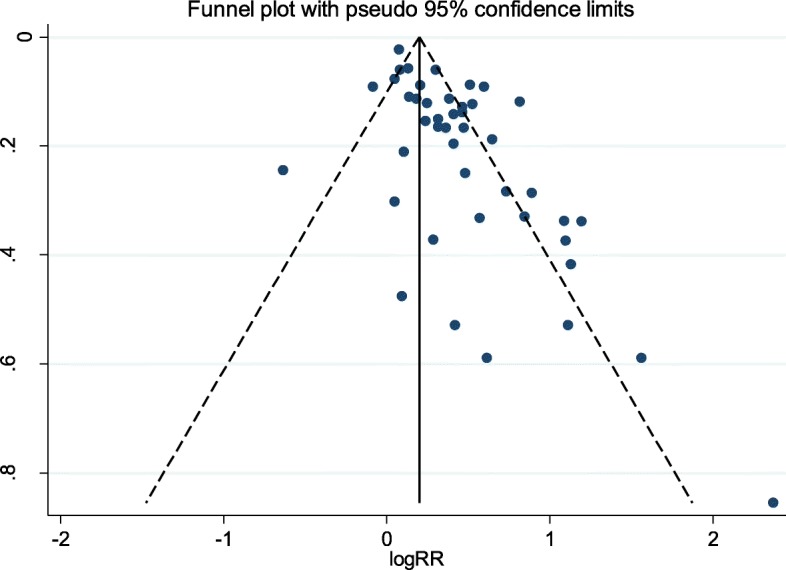
Funnel plots to assessment publication bias

Figure 5 shows funnel plots of CVD mortality. There was publication bias among the studies (the Begg’s *p* = 0.04 and Egger’s test). Using the ‘Trim and fill’ method to adjust for publication bias and random effects model showed 62 arms with pooled results HR = 1.19 (CI:1.09–1.30).

## Discussion

The present meta-analysis of cohort studies revealed that there is a strong relationship between SUA levels and risk of CVD mortality. Moreover, the pooled HR from the random-effects dose-response model indicated that this positive association is stable when SUA is greater than 6 mg/dL. Although the exact mechanism of the relationship between SUA and CVD mortality risk has not yet been elucidated, previous studies have suggested some possible explanations..

An experimental study has reported that using an inhibitor of uricase can elevate blood pressure in rats by activating the renin-angiotensin system and inhibition of nitric oxide synthase [43]. Moreover, it has been shown that patients with hypertension and hyperuricemia had higher carotid intima media thickness in comparison to patients without hyperuricemia [44]. Furthermore, it has been reported that blood atherosclerotic platelets consist of a great amount of UA and higher levels of SUA can simply boost thrombus development which may lead to slow coronary flow (SCF) [45]. In contrast, UA may have some anti-proliferative influence on the endothelium or can damage the process of nitric oxide production [16].

It has been suggested that UA can significantly slow down coronary flow by promoting the calcification of coronary arteries [45, 46]. In addition, higher levels of SUA may induce oxidative stress by oxidation of low-density lipoprotein cholesterol which may lead to SCF [45]. Another possible mechanism might be explained by the effects of hyperuricemia in induction of crystal shaping on vascular walls that impair endothelial and smooth muscle function leading to atherosclerosis by renin-angiotensin system activation [43, 47]. Even, crystals of urate have several noxious effects that activate neutrophilsand macrophage cells to set proteases free and stimulate the coagulation cascade [48].

The main strength of this meta-analysis was the application of cohort studies containing over one million participants, and subsequent dose-response analysis. This study has some possible limitation. The heterogeneity in the study populations is the main limitation of this study. Moreover, the duration of follow-up in our included studies differed. Further, there is an insufficient number of randomized clinical trials to confirm the effects of decreasing SUA levels on CVD deaths; however, our results suggest higher levels of SUA is an independent risk factor associated with CVD mortality. Furthermore, although we searched the literature extensively, we did not explore grey or unpublished literature to limit the possibility of publication bias.

## Conclusion

In conclusion, our analysis indicates a positive dose-response association between SUA and CVD mortality risk; however, further clinical trials are needed to confirm these findings and to determine the possible cause-effect relationship.

## Data Availability

All data are presented within the manuscript. Raw data can be available by corresponding author per request.

## Abbreviations

CI: Confidence interval
CVD: Cardiovascular diseases
HR: Hazard ratio
SCF: Slow coronary flow
SUA: Serum uric acid
UA: Uric acid
